# Parturition Arrested by a Large Calculus in the Bladder

**Published:** 1856-02

**Authors:** 


*Parturition arrested by a large Calculus in the Bladder.*—Mr. Erichsen exhibited to the Pathological Society of London, Nov. 20, 1855, a large calculus from the female bladder, which had arrested parturition, from a woman twenty-five years of age, who was confined with her first child in

October. The accoucheur found a large tumor obstructing the passage of the head, and he was compelled to perform craniotomy in order to effect delivery. On examination the tumor was found to be a vesical calculus, which had never previously been detected or suspected, although the patient had suffered from childhood from irritability of the bladder. She was sent up to Mr. Erichsen from the country three weeks after delivery. On examination, a large calculus was found bulging the posterior wall of the bladder into the vagina. The parts were very tender. She suffered the most excruciating and constant agony, and wished that the stone might at once be removed. She was kept in hospital for a few days, and then the calculus was cut out through the vagina, by the vesico vaginal operation. The stone weighed five ounces and a half, and measured eight inches in the long and six inches in the short circumference. The patient went on well for about eight days, when she suddenly died exhausted. On *post-mortem* examination, extensive disease of the kidneys was found; the right was covered into a mere cyst, and the left was in a state of chronic pyelitis, with great dilatation of the ureter. The case is interesting—first, on account of the very large size of the stone; secondly, from its obstructing parturition, an occurence of which he (Mr. Erichsen) had not been able to find another instance; thirdly, from the absence of symptoms of sufficient urgency to lead to its detection until after the parts had been dilated, and probably bruised, by the efforts during delivery.

In reply to a question from Mr. Holmes, whether there had been any objection to the high operation, Mr. Erichsen said he had considered that, but preferred the vesico-vaginal operation, as being more likely to relieve the condition of the bladder in a shorter time, and secure a depending opening. He was afraid to submit the woman to any operation that would give inconvenience, as the lower fundus of the bladder was very irritable and thin.

